# Coexistent Ipsilateral Internal Carotid Artery Occlusion and Cerebral Venous Thrombosis in Hepatitis C

**DOI:** 10.1177/2324709617750179

**Published:** 2018-01-09

**Authors:** Karan Seegobin, Somphanh Khousakhoun, Ryan Crooks, Satish Maharaj, Cherisse Baldeo

**Affiliations:** 1 University of Florida, Jacksonville, FL, USA

**Keywords:** hepatitis C, carotid artery occlusion, cortical vein thrombosis

## Abstract

A 58-year-old male, known to have hepatitis C virus (HCV), presented with intermittent headaches and left-sided sensorimotor symptoms. There were no focal neurological deficits on examination. Electrocardiogram was unremarkable. Computed tomography angiography head and neck displayed extracranial right internal carotid artery occlusion. Magnetic resonance imaging showed right cortical vein thrombosis, with hemorrhagic infarction. Echocardiography with bubble study was unremarkable. Hypercoagulable workup was significant for protein S deficiency. He was treated with warfarin for 6 months. Repeat protein S levels remained low 9 months later. The coexistence of arterial and venous thrombotic events gives rise to a limited differential. In this case, it may be related to chronic HCV infection. The underlying pathogenesis is not clear; however, it is possible the patient had chronic high-grade internal carotid artery stenosis, which occluded leading to his presenting symptoms. The cortical vein thrombosis is likely an incidental finding here. The extent by which HCV contributed to the cerebral thrombosis and carotid artery occlusion in our case is not clear; however, the hypercoagulable and atherosclerotic properties of the virus cannot be disregarded. The virus can promote carotid atherosclerosis and cerebral venous thrombosis as well as other venous and arterial thromboembolic events. Furthermore, HCV is associated with impaired venous flow and procoagulant properties, which can fuel a hypercoagulable state. Also of note cirrhosis is associated with protein S deficiency. We recommend considering an underlying hypercoagulable state including both arterial and venous thrombosis in HCV infection.

## Background

While cerebral venous thrombosis is reported in the setting of hepatitis C, and hepatitis C virus (HCV) is considered a potential risk factor for ischemic stroke,^1^ from our literature search we have not found any cases with both cerebral arterial and venous thrombosis present in the setting of hepatitis C infection. With search terms “cortical vein thrombosis” and “arterial occlusion” on PubMed, coexistence of the cerebral artery occlusion and cortical vein thrombosis is described infrequently in the literature.^2^ In the presence of hepatitis C, with its atherogenic and prothrombotic properties, we postulate that it may have had a role in the development of his complete carotid artery occlusion and cortical vein thrombosis.^3,4^ However, it is likely in this case that the cortical vein thrombosis may have been an incidental finding. We describe a case of ipsilateral arterial and venous thrombosis in a patient with chronic hepatitis C who was found to have low protein S levels.

## Case Presentation

We describe the case of a 58-year-old male who complained of intermittent headache and dizziness for a duration of 4 days prior to his presentation. Headaches were intermittent and associated with weakness and numbness on the left side. The episodes lasted 1 minute and completely resolved prior to his presentation to the emergency department. There was no loss of consciousness or head trauma. The medical history was significant for chronic hepatitis C. He has a history of using alcohol and a 40-pack-year history of cigarette smoking. He had no allergies, and he had not been on any medications. Furthermore, there was no history of herbal or illicit drug use. His family history was noncontributory.

On examination, his blood pressure was 131/85 mm Hg, pulse rate 67/minute, respiratory rate 18/minute, temperature 36.6°C, oxygen saturation 99% on room air, and body mass index 23.87. He was alert and oriented in time, place, and person, and he had a Glasgow Coma Scale score of 15/15. Pupils were equal round and reactive to light. There were no cranial nerve deficits. He had normal tone and power 5/5 in both upper and lower limbs. Plantars were down going and reflexes 2+ throughout. Coordination was normal. Heart sounds S1 and S2 were normal without associated murmurs, gallops, and rubs. Other aspects of the physical examination were normal.

## Investigations

His chest X-ray showed normal lung fields. Electrocardiogram was regular with a normal sinus rhythm at a rate of 51/minute. Hematologic data showed that the hemoglobin level and white blood cell count were within normal limits. The random blood glucose was 106 mg/dL, and HbA1c 5.9%. The basal metabolic profile, lipid profile, thyroid function test, vitamin B_12_, and folate were within normal limits, and the rapid plasma reagin was negative. His hypercoagulable workup was significant for protein S deficiency 43 (60-145); other parameters of this screen (protein C, antithrombin III, factor V Leiden mutation, antiphospholipid antibodies including anticardiolipin antibodies, lupus anticoagulant, and β-2-glycoprotein-1 antibodies, fibrinogen, and homocysteine) were reported within normal limits. Hepatitis C antibody was positive. Hepatitis C genotype 1b was present and HCV NAA Quantitative test showed 3 190 000 IU/mL. Hepatitis B and A antibodies and hepatitis B surface antigen were negative. His hepatic function panel showed the following: aspartate aminotransferase 44 U/L (14-33 U/L), alanine aminotransferase 46 U/L (10-42U/L), *alkaline phosphatase* 71 U/L (40-129 U/L), albumin 4.2 g/dL (3.8-4.9 g/dL), total protein 7.8 g/dL (6.5-8.3 g/dL), direct bilirubin 0.2 mg/dL (0-0.2 mg/dL), indirect bilirubin 0.6 mg/dL (0.2-0.9 mg/dL), and total bilirubin 1.4 mg/dL (0.2-1.0 mg/dL). Coagulation parameters showed normal international normalized ratio 1.1 (0.9-1.1), activated partial thromboplastin time 75 seconds (24-34 seconds), and partial thromboplastin time 4.5 seconds (11-14.3 seconds). His antinuclear antibody and cryoglobulin levels were reported within normal limits. Urine toxicology screen was negative. Abdominal ultrasound with Doppler showed a cirrhotic liver without splenomegaly.

**Figures 1 and 2.**
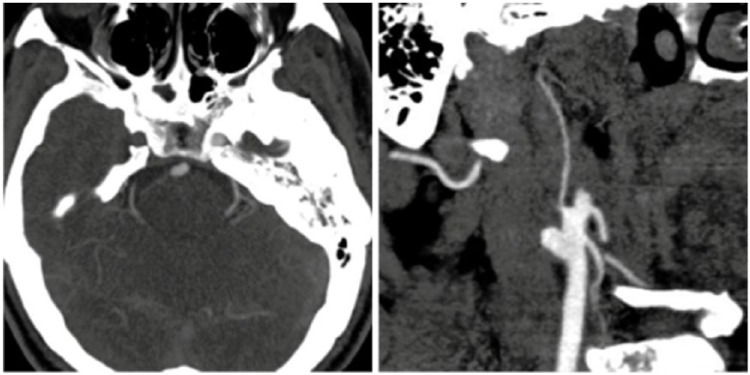
Computed tomography angiography shows loss of enhancement in the right internal carotid artery axial section (Figure 1) and oblique section (Figure 2).

Computed tomography brain noncontrast showed no infarct or hemorrhage, with normal grey-white matter differentiation. Computed tomography angiography head and neck displayed extracranial right internal carotid artery (ICA) occlusion (Figures 1 and 2).

**Figure 3 and 4.**
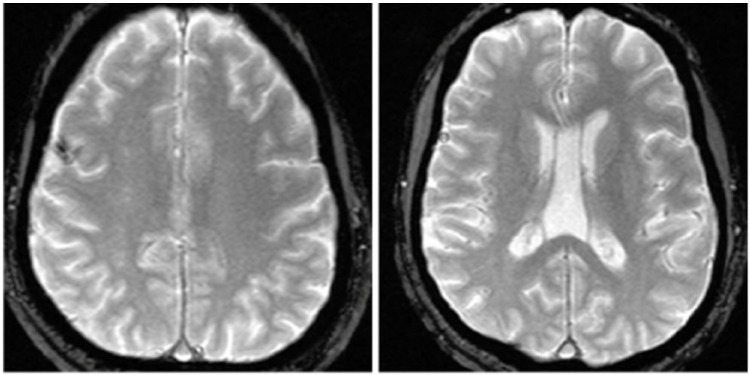
Axial section T2 Flare MRI image showing infarction of the right posterior frontal cortex (Figure 3) and cortical vein thrombosis in right posterior frontal (Figure 4).

He was admitted and started on aspirin 325 mg then 81 mg daily, and magnetic resonance imaging (MRI) of the brain was ordered. Vascular surgery consult did not opt for surgical intervention due to the vessel being occluded. They recommended continuation of aspirin daily. MRI of the brain showed cortical vein thrombosis with small hemorrhagic infarction (Figures 3 and 4). Echocardiography with bubble study showed ejection fraction of 60%, without any thrombus, and no patent foramen ovale.

## Treatment

Following the diagnosis of the cortical vein thrombosis, he was started on warfarin and followed-up in the outpatient setting and given the hepatitis B vaccine. The patient was not willing for treatment of his hepatitis at the time.

## Outcome and Follow-up

The patient had no further symptoms during his hospital stay and was started on warfarin for a duration of 6 months and followed up in the outpatient setting. He remained asymptomatic thereafter. Approximately 9 months after his presentation his repeat protein S levels were still low at 43.

## Discussion

The coexistence of cerebral arterial and venous sinus occlusion was first described by Barnett and Hyland in 1953.^5^ They found 3 of 39 autopsied cases (7.7%) of noninfective intracranial venous thrombosis were associated with cerebral arterial occlusion.^5^ However, reports of nonfatal cases with such a combination have been uncommon.^5^ Melamed et al in 1976 described 2 cases of aseptic cavernous sinus thrombosis after intracranial ICA occlusion.^5^ The underlying etiology for coexisting arterial and venous thrombotic events can be challenging; in this case, it may be related to chronic HCV infection.

Cerebral venous thrombosis can result from hypercoagulable states, obesity, trauma, intracranial and local infections, pregnancy and purperium, and the use of oral contraceptives.^6^ Frequently, the cause of cerebral venous thrombosis is multifactorial, and in up to 35% of patients no contributing factors can be identified.^6^ On the other hand, hypertension, diabetes, hyperlipidemia, and smoking are among the risk factors for arterial thrombosis.^7^ There is also a 1.5- to 3-fold increased venous thrombotic risk in individuals who have been exposed to traditional arterial thrombotic risk factors like diabetes, hypertension, and dyslipidemia.^7^ Furthermore, it appears from the literature that patients with arterial thrombosis have from 1.2-fold to more than 4-fold increased risk of developing subsequent venous thrombosis.^7^ The coexistence of arterial and venous thrombotic events gives rise to a limited differential, and in this case, there is the rare coexistence of cerebral arterial and venous thrombus, which may be related to chronic HCV infection.

The order of occurrence of either thrombosis is uncertain from the history of our patient. It is possible he may have had chronic high-grade ICA stenosis, which occluded and led to the presenting symptoms and fluctuating signs. Additionally, HCV and tobacco use could have contributed to the development of this patient’s right ICA occlusion. HCV has been described in the literature to promote the occurrence and progression of carotid atherosclerosis and increases the risk of both venous and arterial thromboembolic events among patients with the infection.^3,8^ Ambrosino et al demonstrated that HCV-RNA was present within carotid plaques.^9^ Although not fully proven, these findings suggest that active local infection of HCV may have an impact on the pathology of arterial wall cells.^9^

Regarding his cortical vein thrombosis, again the role of hepatitis C and the presence of low protein S levels cannot be disregarded. Chronic HCV infection has been considered as a rare cause of cerebral venous thrombosis.^1^ Though reported mechanisms of cerebral venous thrombosis in patients with hepatitis B and C are not fully understood, there is growing evidence that these viruses alone or in combination with a series of other factors may shift the delicate procoagulant thrombolysis balance toward thrombosis.^1^ It is speculated that the HCV envelope protein has a procoagulant activity and that the virus genome encodes serine proteases that could also act as procoagulant.^1^ As protein C, protein S, antithrombin III, and plasminogen are produced by the liver, their levels may decrease in patients with chronic liver disease resulting in a prothrombotic state.^10,11^

Additional mechanisms by which hepatitis C contributes to venous thrombosis include impaired venous flow and vasculopathy, the presence of anticardiolipin and antiphospholipid antibodies, higher thrombin generation rates, and the prevalence of cryoglobulinemia.^12^

From our literature search, there were 2 reports with cerebral venous thrombosis, in the setting to hepatitis A and C, respectively, with several reports of hepatitis B and cerebral venous sinus thrombosis.^1,13,14^

In our case, hepatitis C and low protein S levels could have contributed to our patient’s cerebral vein thrombosis; however, it may have just been an incidental finding.

The family history was negative for thrombosis or protein S deficiency and as a result an acquired protein S deficiency was considered in this patient, probably secondary to chronic hepatitis C. On the other hand, cirrhosis may be an underlying cause of his low protein S levels.^15^

Some reports indicate that decreased arterial flow can lead to venous stasis and thrombosis.^4^ This has been shown in reports of retinal vein thrombosis occurring in the setting of stenosis of the ICA.^4^ However, there is insufficient data on these mechanisms; whether our patient’s complete arterial occlusion led to cortical thrombosis could not be proven.

## Conclusion

Physicians should consider underlying prothrombotic states in patients with coexistent cortical thrombosis and carotid artery occlusion. Hepatitis C should be among this list of differentials.
